# Hyperactivation of mTORC1 signaling mediates folliculin deficiency–induced pulmonary cyst formation in Birt-Hogg-Dubé syndrome

**DOI:** 10.1172/JCI194300

**Published:** 2026-02-16

**Authors:** Ke Cao, Hui Chen, Ling Chu, Hong-Jun Wang, Jianhua Zhang, Yongfeng Luo, Joanne Chiu, Damir Khabibullin, Nicola Alesi, Matthew E. Thornton, Brendan H. Grubbs, Ali Ataya, Nishant Gupta, Francis X. McCormack, Kathryn A. Wikenheiser-Brokamp, Elizabeth P. Henske, Wei Shi

**Affiliations:** 1 Division of Pulmonary, Critical Care and Sleep Medicine; Department of Internal Medicine; University of Cincinnati College of Medicine, Cincinnati, Ohio, USA.; 2 The Saban Research Institute, Children’s Hospital Los Angeles, Keck School of Medicine, University of Southern California, Los Angeles, California, USA.; 3 Division of Pulmonary and Critical Care Medicine, Brigham and Women’s Hospital and Harvard Medical School, Boston, Massachusetts, USA.; 4 Division of Maternal Fetal Medicine, Department of Obstetrics and Gynecology, Keck School of Medicine, University of Southern California, Los Angeles, California, USA.; 5 Division of Pulmonary, Critical Care and Sleep Medicine; Department of Internal Medicine; College of Medicine, University of Florida, Gainesville, Florida, USA.; 6 Division of Pathology & Laboratory Medicine and Perinatal Institute Division of Pulmonary Biology, Cincinnati Children’s Hospital Medical Center, Cincinnati, Ohio, USA.; 7 Department of Pathology & Laboratory Medicine, University of Cincinnati College of Medicine, Cincinnati, Ohio, USA.

**Keywords:** Cell biology, Development, Pulmonology, Mouse models, Tumor suppressors

## Abstract

Germline loss-of-function folliculin (*FLCN*) gene mutations cause Birt-Hogg-Dubé (BHD) syndrome, in which pulmonary cysts are present in up to 90% of the patients. The pathogenic mechanisms underlying lung cyst development in BHD are almost entirely unknown because of the limited availability of BHD patient lung samples and the lack of authentic BHD lung disease models. We generated lung mesenchyme–specific and lung epithelium–specific *Flcn*-knockout mice using a Cre/*loxP* approach. We found that deletion of *Flcn* in lung mesenchymal cells, but not in lung epithelial cells, resulted in alveolar enlargement starting from early postnatal life, with evidence of cyst formation in adult mice, resembling the pulmonary disease in human BHD. These changes were associated with increased mechanistic target of rapamycin complex 1 (mTORC1) activity in the lungs of both patients with BHD and *Flcn*-knockout mice. Attenuation of mTORC1 activity by knocking out Raptor gene (*Rptor*) or pharmacologic inhibition using rapamycin substantially rescued the pulmonary pathology caused by *Flcn* deletion in mice. Taken together, these human and mouse data support a model in which mTORC1 hyperactivation drives pulmonary cystic pathology in BHD.

## Abstract 2

mTORC1 hyperactivation is found in Birt-Hogg-Dube patient’s cystic lungs, and its role in lung cyst formation is validated using a novel pre-clinical model of BHD

## Introduction

Germline loss-of-function mutations in folliculin (*FLCN*) gene cause an autosomal dominant disorder Birt-Hogg-Dubé syndrome (BHD), which is clinically characterized by facial skin fibrofolliculomas, renal cell carcinoma, cystic lung disease, and pneumothorax (1). A health care system–based study has revealed that the prevalence of germline *FLCN* mutations is about 1 in 3,500, much higher than previously suspected (2). Multiple bilateral pulmonary cysts are present in up to 90% of BHD cases (3), representing a contrast with the proliferative lesions in the kidney and skin. A recent longitudinal study revealed that the pulmonary cysts in BHD tend to slowly increase in size during adulthood accompanied by a gradual decline in pulmonary function (4). More than one-third of patients with BHD experience at least 1 spontaneous pneumothorax in their lifetime, with a very high rate (>70%) of recurrence (3, 5). Although loss-of-function *FLCN* mutations are known to be the cause of BHD, the pathogenic mechanisms underlying cystic lung pathology in BHD remain poorly understood. Mechanistic target of rapamycin complex 1 (mTORC1) plays a role in BHD renal disease, but mTORC1 regulation in BHD appears to be highly context dependent, with hyperactivation of mTORC1 in some models and hypoactivation in other models (6, 7). Lung biopsy is not informative in BHD, as there are no pathognomonic histopathological findings. While multiple mouse models of BHD-associated renal disease have been developed (8–11), in vivo models that authentically recapitulate BHD-associated cystic pulmonary disease have not been developed, and the pathogenesis of lung cysts in BHD remains incompletely understood (12). These challenges highlight the importance of developing an animal model of BHD that can be used to test key hypotheses related to the initiation and progression of pulmonary cyst formation.

Homozygous deletion of *Flcn* in mice leads to embryonic lethality, and cystic lung pathology has not been reported in mice with heterozygous *Flcn* deletion (8, 11). Previously, we deleted *Flcn* in mesoderm-derived mesenchymal cells using the *Dermo1-Cre* driver. This resulted in abnormalities in multiple organs in early postnatal life and premature death (<1 month of age) (13), preventing analysis of the progression of the pulmonary lesions into adulthood. Moreover, these Dermo1-Cre/*Flcn*^fl/fl^ mice have retardation of whole-body development, including a small chest cavity, potentially confounding conclusions related to the roles of FLCN in lung alveolar development. There is a critical unmet need to develop an authentic animal model of BHD to identify the mechanisms underlying cyst formation and to develop strategies for prevention and therapy.

In this study, we compared lung tissues from patients with BHD with healthy controls and found increased phosphorylation of S6 ribosomal protein (pS6; a marker of mTORC1 activation) in alveoli. To develop a mouse model of BHD-associated lung pathology, we selectively deleted *Flcn* specifically in lung mesenchymal cells using a *Tbx4-rtTA/TetO-Cre* driver. These mice exhibit defective postnatal lung alveolarization, which is evident at P7 to P14. The lung pathology progresses as the mice age, with bilateral pulmonary cyst formation resembling human BHD lesions by the age of 4 months. In contrast, *Flcn* deletion in lung epithelial cells alone did not result in any alveolar enlargement, and there were no synergistic or additive effects when *Flcn* was deleted in both epithelial and mesenchymal compartments compared to the mice with lung mesenchymal *Flcn* deletion alone. Deletion of Raptor gene (*Rptor*), a key component of mTORC1 (14), along with *Flcn* in lung mesenchyme, markedly reduced alveolar enlargement and cyst development in adult *Flcn*/*Rptor* double knockout mice, though modest alveolar enlargement was still observed, suggesting that hyperactivation of mTORC1 is one of the mechanisms underlying pulmonary pathology in BHD. Consistently with the *Flcn/Rptor* double knockout mouse data, treatment of the *Flcn*-knockout mice with rapamycin partially rescued the alveolar enlargement and cyst formation. These studies suggest that loss of mesenchymal FLCN leads to alveolar defects in BHD that is caused by both mTORC1-dependent and -independent pathways.

## Results

### Increased mTORC1 activation in human BHD lung specimens.

**Figure 1:**
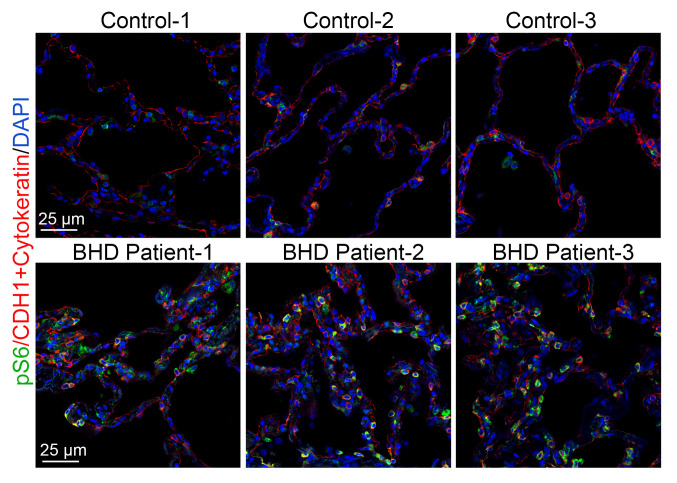
Increased pS6 in human BHD lung tissue specimens. Lung tissues from 3 patients with BHD patients and 3 non-BHD healthy donors were immunostained with anti-pS6 antibody (shown in green). Epithelial cells were marked by CDH1 (or E-cadherin) and cytokeratin immunostaining (shown in red), and cell nuclei were counterstained with DAPI (shown in blue).

Studies in cultured cells and kidneys with *Flcn* deletion have revealed both increased and decreased mTORC1 activity (10, 15). To assess the state of mTORC1 activity in lung lesions of BHD, we examined pS6, a downstream target of the mTORC1 activation cascade. Interestingly, increased pS6 was detected in the lungs of the BHD lung samples compared with non-BHD controls (Figure 1), shown in both alveolar epithelial (highlighted by CDH1 and cytokeratin immunostaining) and nonepithelial compartments, suggesting that abnormal mTORC1 activation may play a role in the pathogenesis of pulmonary cyst formation in BHD.

### Generating a BHD mouse model by lung mesenchymal cell–specific Flcn deletion.

**Figure 2:**
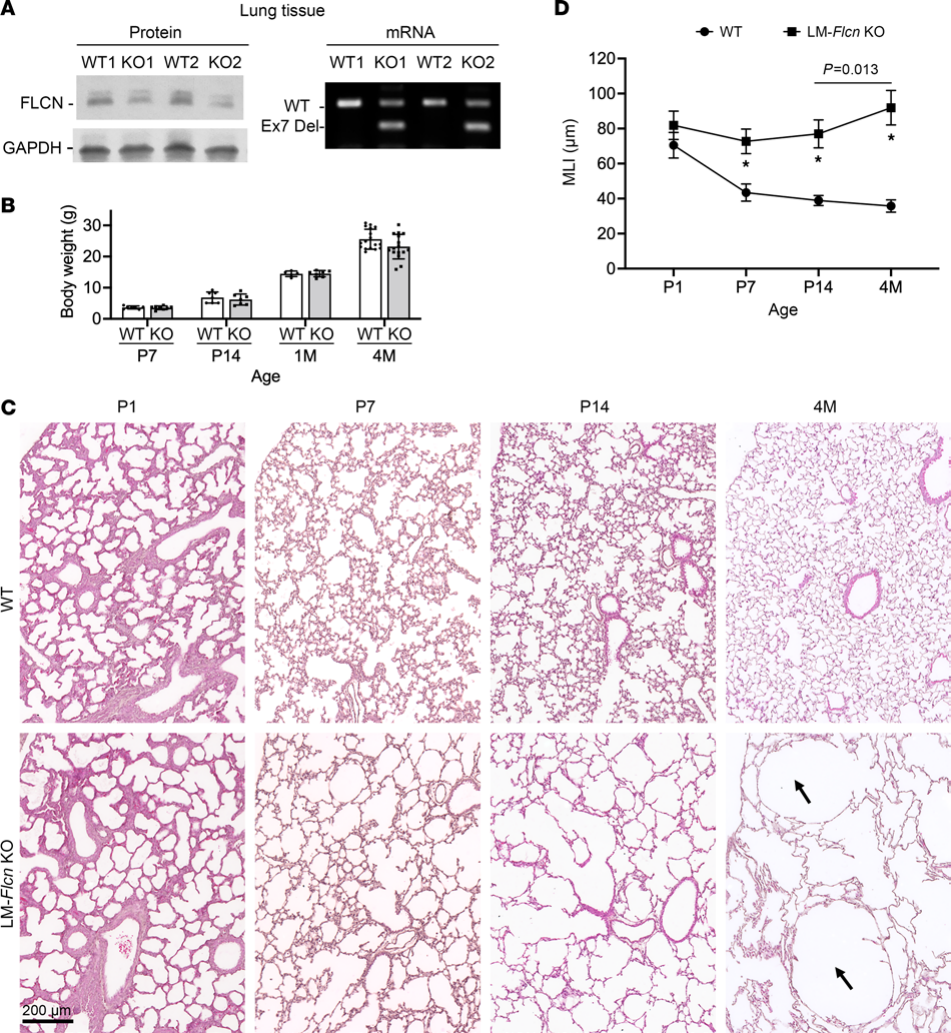
Deletion of *Flcn* in lung mesenchyme results in abnormal alveolar development and disrupted lung homeostasis. (**A**) Validation of Flcn protein reduction and exon 7 deletion (Ex7 Del) in *Flcn* mRNA of the lung mesenchyme Flcn knockout (LM-*Flcn* KO) mouse lung tissues at P14. (**B**) Body weight comparison of LM-*Flcn* KO and littermate WT control mice at 4 time points. (**C**) H&E-stained lung tissue sections at 4 time points, showing progressive alveolar enlargement beginning at P7. Cystic lung lesions in the LM-*Flcn* KO lung at 4 months are indicated with arrows. (**D**) Quantification of alveolar size by measuring mean linear intercept (MLI) in LM-*Flcn* KO and WT mice at the indicated time points (*P < 0.001). The LM-*Flcn* KO mice have increased MLI from P14 to 4M (*P* = 0.013 using 2-tailed independent-sample *t* test). Numbers of mice: P1 WT = 4, P1 KO = 3, P7 WT = 7, P7 KO = 7, P14 WT = 5, P14 KO = 6, 4M WT = 8, 4M KO = 8. All images were taken at the same magnification.

Mouse mesodermal deletion of *Flcn* using *Dermo1-Cre* driver results in multiple organ abnormalities and death prior to P30 (13), limiting the utility of this model to study the formation and progression of pulmonary cysts in BHD. We generated a lung mesenchyme–specific *Flcn*-knockout mouse model, *Tbx4-rtTA*/*TetO-Cre*/*Flcn*^fl/fl^, in which lung mesenchyme–specific *Flcn* gene knockout is induced by administering doxycycline during fetal lung development from embryonic day (E) 6.5 to P1 (16). Mesenchymal *Flcn* deletion resulted in lower levels of the FLCN protein in lung tissue lysates, and *Flcn* exon 7 truncation was validated at the mRNA level (Figure 2A). Increased mTORC1 activity, as measured by elevated phosphorylation of the mTORC1 targets S6 and 4E-BP1, was detected in primary lung mesenchymal cells isolated from the *Tbx4-rtTA*/*TetO-Cre*–driven lung mesenchyme–specific *Flcn*–conditional knockout mice (hereafter referred as LM-*Flcn* KO mice, Supplemental Figure 1; supplemental material available online with this article; https://doi.org/10.1172/JCI194300DS1). The LM-*Flcn* KO mice had normal body weight comparable to their WT controls, which included all littermates without *Flcn* deletion (Figure 2B). These LM-*Flcn* KO mice survived to adult ages without observable physical differences compared to the WT controls. Thus, this model allows us to study the evolution of lung pathology from the neonatal stage to adulthood in the absence of extraneous and potentially confounding factors that may negatively affect lung growth and homeostasis, such as the reduction in chest cavity size and attenuated musculoskeletal growth that were present in the Dermo1-Cre–driven *Flcn*-knockout mouse model.

The histopathology of the LM-*Flcn* KO lungs was examined from lung development stages (<1 month of age) to the postdevelopment period (4 months). While lung saccular structures were comparable between newborn (P1) LM-*Flcn* KO mice and their WT littermates, a marked reduction in alveolar formation was observed in LM-*Flcn* KO lungs from P7 to P14, when the lungs of WT mice underwent normal secondary alveolarization (Figure 2C). This was quantitatively validated by measuring average alveolar size (mean linear intercept, MLI) (Figure 2D), revealing that the alveolar size did not change appreciably from P1 to P14 in LM-*Flcn* KO lungs, in striking contrast with the substantial reduction in WT controls because of alveolar growth–related subdivision of peripheral saccular spaces. In adult (4-month, 4M) mice, the alveoli were further enlarged in LM-*Flcn* KO mouse lungs with multiple bilateral, cyst-like lesions, particularly in the locations adjacent to the pleural membrane, as observed in human BHD, where the mechanical stress placed on the lung parenchyma during respiration is the greatest (17). This was further supported by striking increases in the MLI from P14 to 4M in LM-*Flcn* KO lungs (*P* = 0.013, *n* ≥ 6). This suggests that additional loss of preformed alveolar septal structures contributes to cyst formation and progression in adult LM-*Flcn* KO mice.

### Mesenchymal FLCN is essential for both alveolar growth and alveolar maintenance.

**Figure 3:**
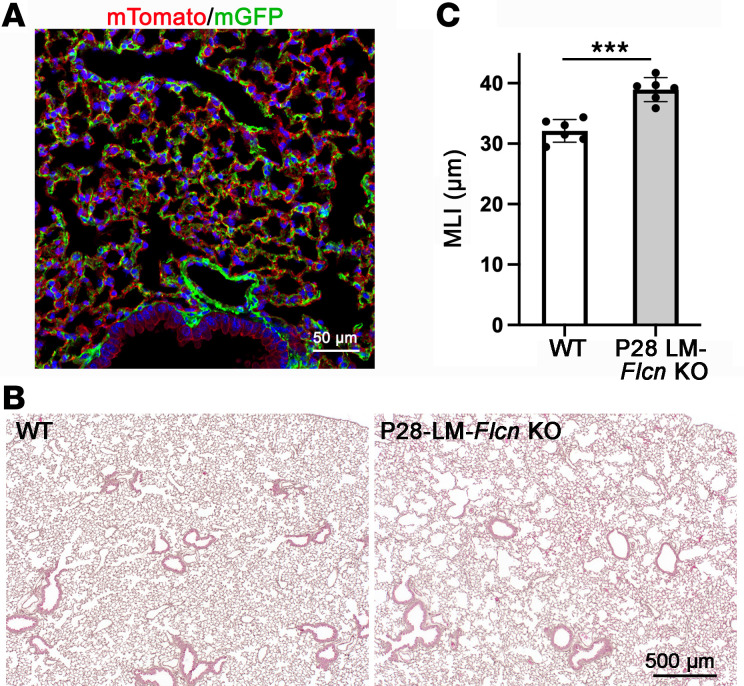
Abrogation of lung mesenchymal *Flcn* after completion of lung development results in moderate alveolar enlargement. (**A**) Validation of Tbx4-rtTA–driven Cre expression in lung mesenchyme after completion of alveolar development (P28–P60). mGFP expression resulting from Cre-mediated floxed-gene recombination was detected in lung mesenchymal cells in the mT-mG reporter lung (*Tbx4-rtTA/TetO-Cre/mT-mG* with doxycycline induction from P28 to P60). (**B**) H&E-stained lung tissue sections at 4 months of age, illustrating alveolar enlargement in P28-induced LM-*Flcn* KO lungs. (**C**) Quantitative comparison of alveolar size (MLI) between P28-induced LM-*Flcn* KO lungs and WT controls (***P < 0.001, *n* = 6), analyzed using 2-sample *t* test.

To determine whether the progressive enlargement of alveoli in adult LM-*Flcn* KO mice is the consequence of abnormal alveolar development or a direct impact of FLCN deficiency in adulthood, we induced *Flcn* deletion after alveolar development by treating the triple-transgenic mice (*Tbx4-rtTA/TetO-Cre/Flcn*^fl/fl^) with doxycycline from P28 to P60. The specificity and efficiency of *Tbx4-rtTA*–driven Cre expression in lung mesenchyme after 1 month of age (postdevelopment stage) were validated by detecting membrane GFP (mG) expression in a Cre-mediated mT-mG reporter mouse line without *Flcn* knockout (*Tbx4-rtTA/TetO-Cre/mT-mG*, Figure 3A). Expression of Cre results in floxed-mT/STOP cassette DNA deletion, thereby allowing downstream mG expression. Therefore, the cells with mG expression are the cells where Cre-mediated gene deletion occurs. Lung histopathology of the P28-induced LM-*Flcn* KO mouse lungs was examined at 4 months of age. Moderate but significant enlargement (*P* < 0.001) of alveoli in peripheral lungs was consistently observed in the P28-induced LM-*Flcn* KO mice (Figure 3B). The average alveolar size in these LM-*Flcn* KO mice was about 20% larger than controls (MLI: 38.9 ± 0.7 μm in P28-induced LM-*Flcn* KO versus 32.1 ± 0.7 μm in WT, *P* < 0.001, Figure 3C). These data indicate that deficiency of FLCN in postdevelopment lung mesenchyme negatively affects lung alveolar homeostasis. The severe cystic lesions in adult mice in which loss of *Flcn* is induced prenatally are therefore predicted to be caused by the combined effects of FLCN deficiency on alveolar development and alveolar maintenance.

### The cellular and molecular impact of FLCN deficiency in lung is age dependent.

**Figure 4:**
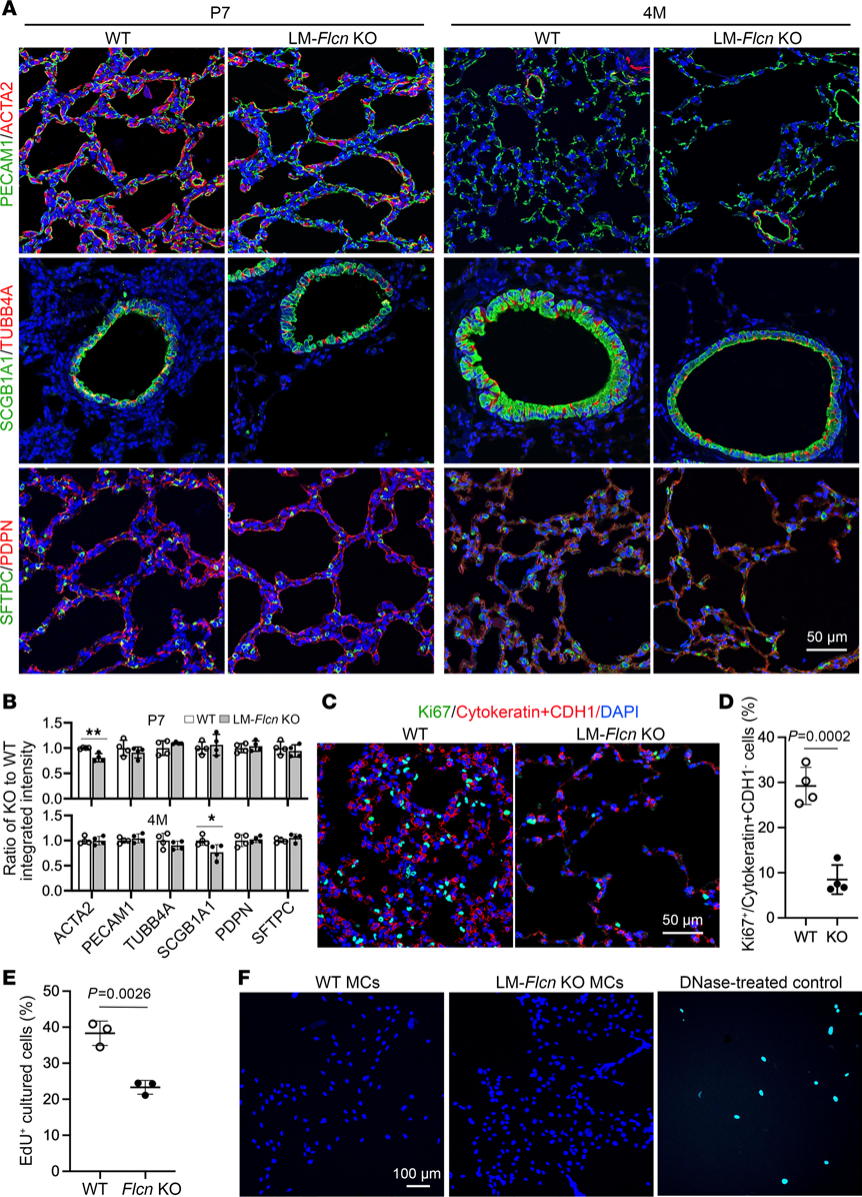
Mesenchymal *Flcn* deletion influences lung cell growth directly and indirectly. (**A**) Lung mesenchymal and epithelial cell differentiation, detected by immunostaining of the indicated cellular markers. (**B**) The integrated intensity of the immunostaining shown in **A** from at least 6 fields per sample and 4 samples per genotype was measured and normalized by the DAPI staining area. The ratios of KO to WT intensity at P7 and 4M are shown (mean ± SD); **P = 0.004, *P = 0.04. (**C**) Mesenchymal proliferation of adult mouse lungs was measured by Ki67 immunostaining for cells that were negative for the combined epithelial markers (cytokeratin and CDH1). (**D**) The Ki67^+^ mesenchymal cells were quantified and compared between LM-*Flcn* KO and WT. (**E**) Comparison of cell proliferation of isolated lung mesenchymal cells in culture from adult WT and LM-*Flcn* KO mice. Experiments were repeated in the cells that were separately isolated from 3 individual mice per genotype. (**F**) TUNEL assay of cultured lung mesenchymal cells (MCs). Normal lung sections pretreated with DNase were used as a positive control for the assay. In **B**, **D**, and **E**, 2-sample *t* test was used. EdU, 5-ethynyl-2′-deoxyuridine.

To understand the mechanisms by which FLCN deficiency causes abnormal alveolar growth and homeostasis, we examined the cellular changes in the lungs during alveolar development versus after alveolar development. Reduction in alveolar myofibroblasts (ACTA2-positive cells, Figure 4A) was detected during alveolar development (P7), while alterations of endothelial cells and epithelial cells in conducting airways and alveoli were not observed at this age. At 4 months of age, alveolar myofibroblasts were nearly absent in both LM-*Flcn* KO and WT control lungs, with no difference detected. Interestingly, at 4 months, proximal airway Club epithelial cells in the LM-*Flcn* KO lungs had a flattened shape and weaker staining for the marker SCGB1A1^+^ relative to the control, while alveolar epithelial cells including type 1 (PDPN) and type 2 (SFTPC) seemed unchanged (Figure 4, A and B). To further elucidate the impact of mesenchymal FLCN deficiency, cell proliferation was evaluated using Ki67 staining. The percentage of Ki67^+^ proliferative cells in nonepithelial cells (negative for cytokeratin and CDH1) was approximately 4-fold reduced in LM-*Flcn* KO lungs compared with WT lungs (Figure 4, C and D). Furthermore, lung mesenchymal cells were isolated and compared between *Flcn* KO and controls. *Flcn* deletion resulted in marked reduction of mesenchymal cell proliferation in culture (Figure 4E), but no differences in cell death were detected by TUNEL assay in the cultured mesenchymal cells from the LM-*Flcn* KO and WT mice (Figure 4F). Interestingly, the isolated *Flcn*-null lung mesenchymal cell colonies stopped growth after passage 3–4 while WT control lung mesenchymal cells remained growing in culture.

**Figure 5:**
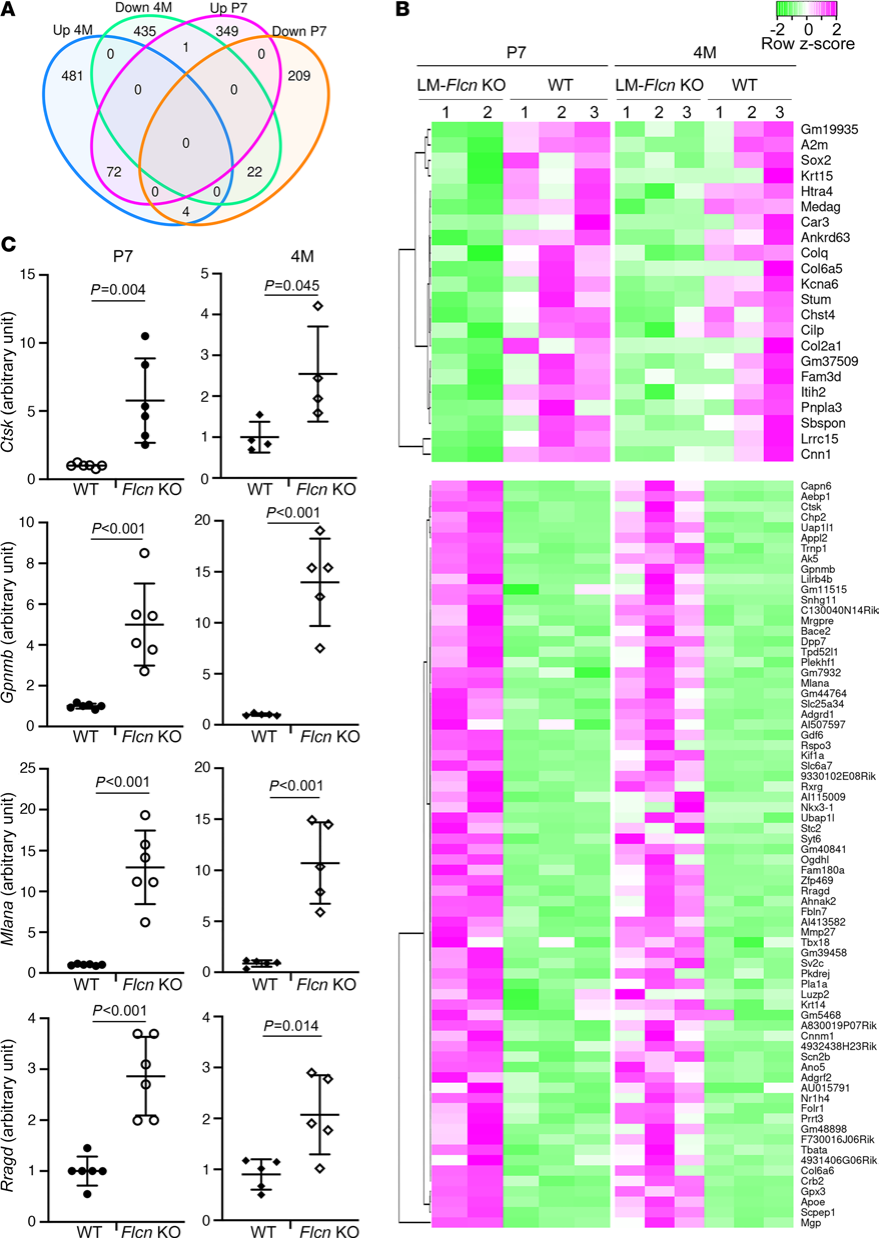
Subsets of genes that are coordinately up- or downregulated in LM-*Flcn* KO lungs at P7 and 4M, including genes related to mTORC1 signaling and lysosome biology. (**A**) Venn diagram summarizing the number of genes that undergo increased and decreased expression (LogFC ≥ 2, FDR < 0.05) at P7 vs. 4M. (**B**) Heatmap of the genes that have consistent changes between P7 and 4M. (**C**) RT-PCR validation of 4 genes (*Ctsk*, *Gpnmb*, *Mlana*, and *Rragd*) related to mTORC1 and lysosomal activities was performed with 2-sample *t* test analysis (*n* = 6 for P7 WT and P7 KO, *n* = 5 for 4M WT and 4M KO).

To further understand the molecular effects of *Flcn* deficiency in this model, we performed bulk RNA-sequencing on LM-*Flcn* KO lungs and WT control lungs at P7 and 4 months of age. Interestingly, the majority of genes with altered expression (log fold-change [LogFC] > 2, FDR < 0.05) between LM-*Flcn* KO and WT lungs were unique to each age group (Figure 5A). The common set of differentially expressed genes (DEGs) between KO and WT lungs that showed consistent expression changes at both ages is shown in Figure 5B. Ingenuity Pathway Analysis was used to identify canonical pathways that were significantly enriched (–log_10_*P* > 1.30, equivalent to *P* < 0.05). The pathways predicted to be activated with positive *Z*-score or inhibited with negative *Z*-score are shown in Supplemental Figure 2. The top pathways that are predicted to be differentially regulated at both P7 and 4M include phagosome formation, G protein–coupled receptor signaling, wound healing, inhibition of matrix metalloproteases, and pulmonary fibrosis. Multiple others, such as WNT/β-catenin, pyrimidine metabolism, SNARE signaling, and melatonin degradation, are predicted to be likely age specific. For example, WNT/β-catenin is increased at 4M but not at P7. Similarly, time-specific DEGs were reported in human kidney proximal tubule cells after *FLCN* knockdown (18). We further examined 4 common DEGs (*Ctsk*, *Gpnmb*, *Mlana*, and *Rragd*), which are related to phagosome/lysosome biology and regulated by mTOR signaling. By RT-PCR, significant increases of their expression were validated in multiple LM-*Flcn* KO lungs at both P7 and 4M (Figure 5C).

### Deletion of lung epithelial Flcn does not negatively influence alveolar structures.

**Figure 6:**
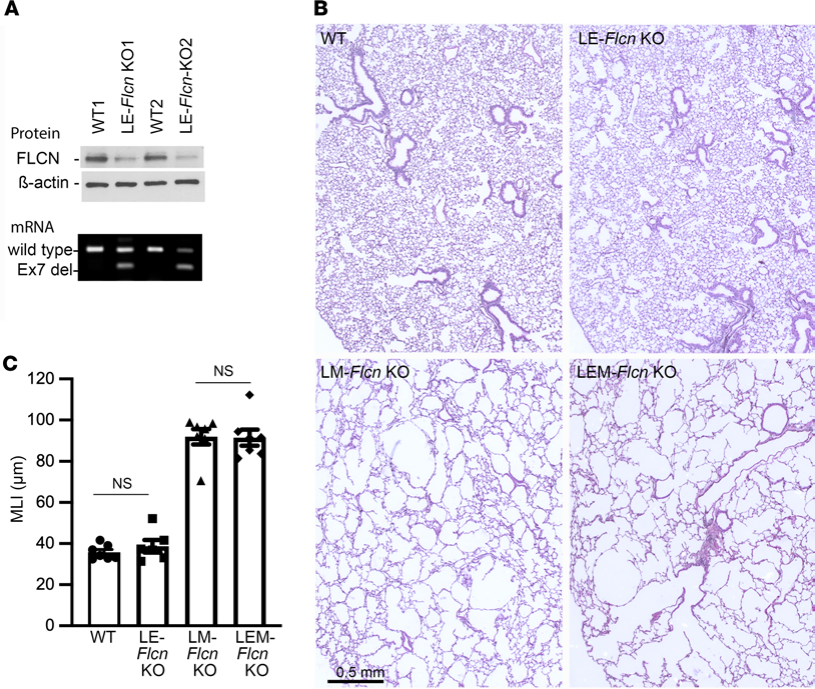
Lack of effect of epithelial *Flcn* deletion in alveolar structure, either alone or in combination with mesenchymal *Flcn* deletion, compared to mesenchymal *Flcn* deletion alone. (**A**) Validation of *Flcn* deletion in LE-*Flcn* KO lungs at the protein and mRNA levels by Western blot and RT-PCR. (**B**) Histopathology of the mouse lungs with the indicated genotypes, shown in H&E-stained lung tissue sections. LEM-*Flcn* KO, *Flcn* knockout in both lung epithelia and mesenchyme. (**C**) Comparison of alveolar size (MLI) between *Flcn* KO mouse lungs by 2-sample *t* test. (*n* = 7 for WT, LM-*Flcn* KO, and LEM-*Flcn* KO, and *n* = 6 for LE-*Flcn* KO.)

Our data provide evidence that *Flcn* deletion in lung mesenchyme is a critical driver of cystic lung pathology in BHD. To understand if *Flcn* deletion in the epithelial compartment might be cooperative with mesenchymal *Flcn* loss in cystic formation, an *Sftpc-rtTA* driver line was introduced into the *Tbx4-rtTA*–driven *Flcn*-knockout mice to generate the quadruple-transgenic mice with genotypes of *Sftpc-rtTA*/*Tbx4-rtTA*/*TetO-Cre*/*Flcn*^fl/fl^. The *Flcn* gene deletion was induced by treating the dams with doxycycline from E6.5 to P1. Lung pathology was compared at 4 months of age among LM-*Flcn* KO, lung epithelial *Flcn* knockout (LE-*Flcn* KO), lung mesenchymal plus epithelial *Flcn* knockout (LEM-*Flcn* KO), and WT littermate control mice (Figure 6A). LE-*Flcn* KO did not have observable changes in alveolar size and structure compared to the controls. The alveolar enlargement in LEM-*Flcn* KO lungs was comparable to LM-*Flcn* KO lungs, as shown by both tissue histopathology and morphometric quantification for MLI (Figure 6, B and C). These data support the hypothesis that deficiency of *Flcn* in lung mesenchymal cells, but not in epithelial cells, drives the cystic lung disease seen in BHD.

### mTORC1 hyperactivation is a key mediator of cystic lung pathology in LM-Flcn KO mice.

**Figure 7:**
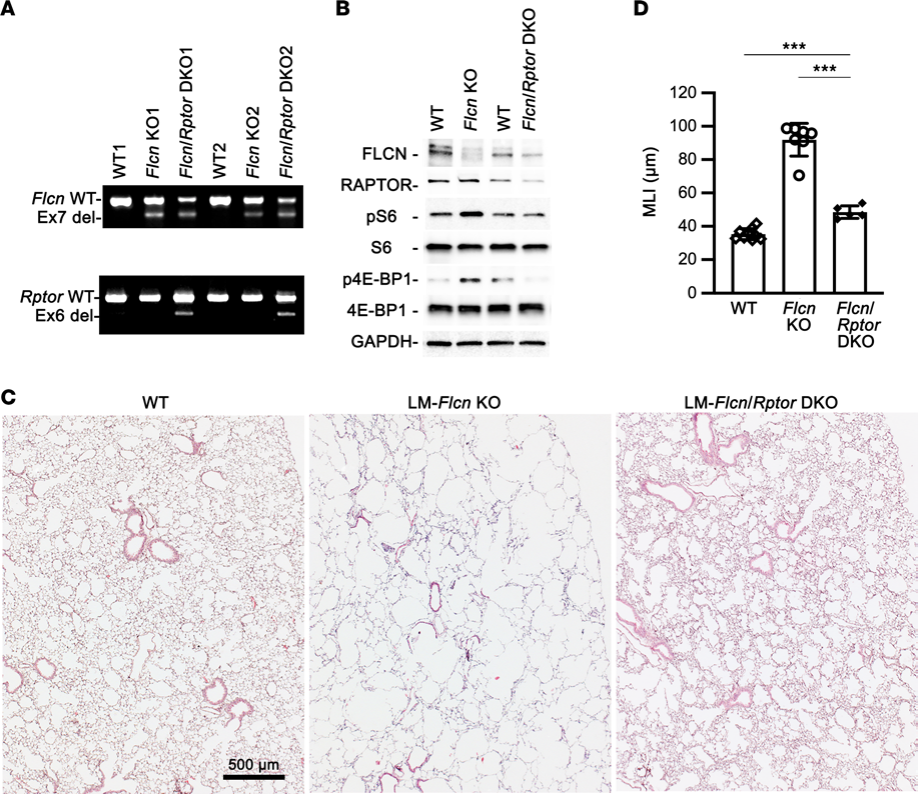
Genetic inactivation of mTORC1 pathway by deleting *Rptor* in lung mesenchymal cells partially rescues the alveolar phenotype in LM-*Flcn* KO lungs. (**A**) Deletion of *Rptor* and/or *Flcn* in the knockout mouse lungs was validated by RT-PCR. (**B**) Assessment of mTORC1 activity in lung tissue lysate was performed by detecting the phosphorylation of S6 (pS6) and 4E-BP1 (p4E-BP1). GAPDH was used as a loading control. (**C**) Lung histopathology in LM-*Flcn* KO mice vs. LM-*Flcn*/*Rptor* DKO mice is shown in H&E-stained lung tissue sections. (**D**) Average alveolar size was quantified and compared among WT, LM-*Flcn* KO, and LM-*Flcn*/*Rptor* DKO mice using a 1-way ANOVA. ***P < 0.001. (Numbers of mice: WT = 9, *Flcn* KO = 7, *Flcn/Rptor* DKO = 5.)

As shown earlier, increased mTORC1 activity was detected in human BHD lung specimens (Figure 1). Hyperactivation of mTORC1, as detected by increased phosphorylation of both ribosomal protein S6 and 4E-BP1, was also seen in LM-*Flcn* KO lung tissues (Figure 7B). mTORC1 comprises mTOR and the essential accessory proteins mLST8 and RAPTOR. To determine whether hyperactivation of mTORC1 signaling mediates *Flcn* deficiency–induced alveolar enlargement and/or destruction in vivo, we blocked mTORC1 activity in the LM-*Flcn* KO lungs by simultaneously deleting *Rptor* and *Flcn* in lung mesenchymal cells (Figure 7A). This lung mesenchyme–specific *Flcn* and *Rptor* double knockout (LM-*Flcn*/*Rptor* DKO, with the genotype of *Tbx4-rtTA*/*TetO-Cre*/*Flcn*^fl/fl^/*Rptor*^fl/fl^) was induced by treating the dams with doxycycline from E6.5 to P1. The mouse lung tissues were harvested and analyzed at adulthood (4–6 months of age). Deletion of lung mesenchymal *Rptor* effectively reversed mTORC1 hyperactivation (Figure 7B). The severe cystic lung phenotype seen in LM-*Flcn* KO mice was substantially rescued in LM-*Flcn*/*Rptor* DKO mice (Figure 7, C and D), as shown by histopathology and morphometric measurement of MLI (91.9 ± 9.8 μm in LM-*Flcn* KO versus 48.5 ± 3.3 μm in LM-*Flcn*/*Rptor* DKO, *P* < 0.001). However, compared with WT littermate control, moderate alveolar enlargement was still present in the LM-*Flcn*/*Rptor* DKO lungs, which was validated by morphometric analysis (MLI: 48.5 ± 3.3 μm in LM-*Flcn*/*Rptor* DKO versus 35.3 ± 3.7 μm in WT controls, *P* < 0.001).

**Figure 8:**
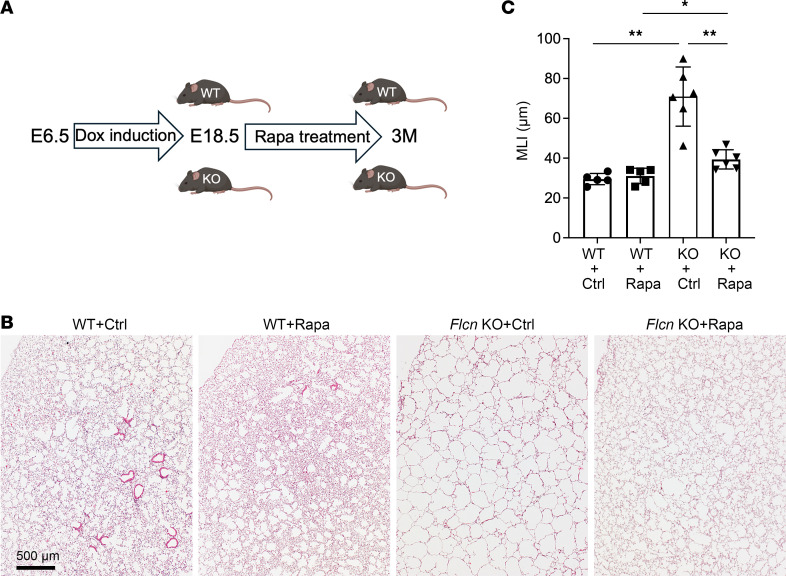
Postnatal rapamycin treatment prevents lung cyst formation in LM-*Flcn* KO mice. (**A**) Schematic diagram for the regimen of rapamycin treatment: intraperitoneal (i.p.) injection (1 mg/kg) every 4 days from E18.5 to P20 and i.p. injection (2 mg/kg) 3 times per week from P20 through 3 months. (**B**) Lung histopathology for the WT and LM-*Flcn* KO mice treated with rapamycin (Rapa) or vehicle control (Ctrl). (**C**) Measurement of alveolar MLI. Significant differences (*P = 0.042 and **P < 0.01) were detected using a 1-way ANOVA. Numbers of mice: WT+Ctrl = 5, WT+Rapa = 5, LM-*Flcn* KO+Ctrl = 6, LM-*Flcn* KO+Rapa = 6.

In addition to genetically blocking mTORC1 specifically in lung mesenchymal cells, we assessed the effect of pharmacologic inhibition of mTORC1 by treating the LM-*Flcn* KO mice with rapamycin beginning after birth (Figure 8A). Consistent with the observation from the genetic deletion of *Rptor* above, rapamycin treatment markedly attenuated alveolar enlargement and cyst formation in *Flcn* KO lung at 4M (Figure 8, B and C, *P* < 0.01), though moderate (~20%) alveolar enlargement was still present in rapamycin-treated *Flcn* KO mice compared with the WT treated with rapamycin (*P* < 0.05). No significant difference in airspace dimensions was detected in WT mice treated with rapamycin versus vehicle control. Taken together, these data from the *Rptor* knockout and the rapamycin treatment models suggest activation of mTORC1 is a major contributor to the lung alveolar abnormality caused by *Flcn* deficiency but is not the only contributor.

## Discussion

The clinical hallmarks of BHD are facial skin lesions, renal cysts and carcinomas, cystic lung disease, and pneumothorax. Germline *FLCN* mutations have been reported in up to 10% of patients with spontaneous pneumothorax who lack other cardinal manifestations of BHD (5, 19–22). Although BHD-related pneumothorax most commonly occurs in the third to fourth decade of life, it has been reported in children as early as 7 years of age (9), and BHD pulmonary cysts have been detected as early as 34 weeks of gestational age (23), when early alveolarization begins. The limited availability of lung tissue from patients with BHD makes it difficult to decipher the exact molecular mechanisms driving pulmonary cyst formation in BHD, highlighting the critical need to develop an in vivo model of BHD that faithfully recapitulates the human pulmonary pathology. Our mouse model with *Flcn* deletion in lung mesenchymal cells from the beginning of lung formation supports a model in which FLCN plays a critical role in early alveolar growth, and FLCN deficiency negatively affects alveolarization, thereby contributing to pulmonary cyst development. Furthermore, FLCN deficiency after alveolar development affects alveolar maintenance in vivo and mesenchymal cell growth in vitro. This suggests that FLCN-null mesenchymal progenitor cells may have reduced capacity for repair and regeneration, resulting in net loss of alveoli over time. Our potentially unique mouse model provides a powerful tool to dissect the mechanisms underlying the roles of FLCN in regulating alveolar development and alveolar homeostasis. In addition, this genetic mouse model will be extremely useful in devising and testing novel therapeutic approaches to prevent the progression of BHD cystic lesions.

We compared the roles of FLCN in lung mesenchymal cells to lung epithelial cells in regulating alveolar growth and maintenance. Deletion of *Flcn* in lung epithelial cells did not directly cause any detectable alveolar structural changes, and simultaneous deletion of lung epithelial plus mesenchymal *Flcn* did not result in more severe pulmonary phenotypes compared to mesenchymal *Flcn* deletion only. It is worth noting that these results differ from a previously published analysis where moderate alveolar enlargement was noted in lung epithelial *Flcn*-knockout mice using the same *Sftpc-rtTA/TetO-Cre* driver line (24). Several factors could account for the discrepancy of these studies, including mouse strain background, difference in doxycycline induction, and lung tissue inflation and fixation techniques. In addition, we observed that mesenchymal *Flcn* deletion indirectly affected conducting airway epithelial differentiation, such as Club cells in adult mice. The mechanisms and pathological impact of such changes remain to be determined.

FLCN can be either a positive or a negative regulator of mTOR signaling, which may be cell type dependent. The roles of mTORC1 in mediating FLCN deficiency–mediated pulmonary lesions are unknown. Treatment of fibrofolliculomas by topical rapamycin did not result in clinical benefit for patients with BHD (25), but rapamycin treatment of *Ksp-Cre*/*Flcn*–knockout mice attenuated kidney cyst formation (26). FLCN has been demonstrated to localize to the subcellular lysosomes, where it acts as a GTPase-activating protein toward RagC/D and as a guanine nucleotide exchange factor toward RagA/B to positively regulate the activity of mTORC1 (15, 27). Although these actions would be predicted to dampen the mTORC1 activity in FLCN-deficient cells compared with control cells (6, 8, 15, 28), elevated mTORC1 activity is found in BHD-associated renal cell carcinoma, *Flcn*-knockout mouse lung, and the resected human lung tissue of a BHD patient with pulmonary cysts (13, 29, 30). To further explore the complex relationship between FLCN and mTORC1 in BHD pulmonary pathology, we used a genetic approach to block mTORC1 activity in lung mesenchymal cells in which *Flcn* is simultaneously deleted. Interestingly, *Flcn* deletion–induced pulmonary phenotypes, including alveolar enlargement and cystic lesions, are largely rescued by the concomitant deletion of the mTORC1 component *Rptor*, suggesting that hyperactivation of mTORC1 is one of the critical pathogenic mechanisms of cystic remodeling in BHD. Furthermore, treatment of the LM-*Flcn* KO mice with mTORC1 inhibitor rapamycin substantially rescues alveolar enlargement and cystogenesis. Therefore, inhibition of mTORC1 hyperactivation may be a therapeutic approach to prevent progression of cystic lung disease in BHD, as has been shown to be the case in another mTORC1-activated cystic disease, lymphangioleiomyomatosis (31). The related mechanisms underlying mTORC1 hyperactivation and abnormal alveolar phenotypes in this model need further investigation. One potential direction is dysregulation of TFEB/TFE3, which are key transcription factors involved in cell autophagy and lysosomal biogenesis. Activation of TFEB along with mTORC1 has been shown to play a critical role in BHD kidney pathology (10). Finally, we note that elucidating the pathogenesis of alveolar structural changes in BHD may have relevance to other lung diseases associated with alveolar dilation and cyst formation in which FLCN may play a critical role, including chronic obstructive pulmonary disease (COPD) (32).

## Methods

### Sex as a biological variable.

Both male and female mice with the desired genotypes were used in all studies, since no phenotypic difference was observed between them.

### Mice.

In our lab (16), a lung mesenchyme–specific *Tbx4-rtTA/TetO-Cre* inducible driver was generated, which was used to cross with the related floxed mouse lines. Floxed *Flcn* (*Flcn*^fl/fl^) mice were provided by Laura Schmidt at National Cancer Institute, Bethesda, Maryland, USA (26). *Sftpc-rtTA* was provided by Jeffrey Whitsett at Cincinnati Children’s Hospital Medical Center (33). mT-mG reporter mice and floxed *Rptor* (*Rptor*^fl/fl^) mice were obtained from The Jackson Laboratory (stock no. 007576 and 013188) (34, 35). All mice were bred into the C57BL/6J strain background and housed in pathogen-free conditions at the animal facilities of Children’s Hospital Los Angeles and University of Cincinnati. Mouse genotypes were determined by genomic DNA PCR using our published methods (13, 16). To induce Cre expression, the mice were fed with doxycycline food (625 mg/kg, TestDiet) and doxycycline water (0.5 mg/mL, Sigma). *Flcn* KO mice were identified based on their DNA genotypes (*Tbx4-rtTA* or *Sftpc-rtTA/TetO-Cre/Flcn*^fl/fl^). Littermates without *Flcn* deletion, including genotypes of *Tbx4-rtTA* or *Sftpc-rtTA/Flcn*^fl/fl^, *TetO-Cre*/*Flcn*^fl/fl^, and *Flcn*^fl/fl^, were designated as WT controls. Deletion of *Flcn* was further validated at the mRNA and protein levels. By RT-PCR, deletion of *Flcn* exon 7 (92 nucleotides) or *Rptor* exon 6 (176 nucleotides) was confirmed using primer pairs (5′-GATGACAACTTGTGGGCGTGTC-3′ and 5′-ACAGGCTGAGAGAAAGGATGAC-3′) and (5′-CGGGTCCTTTTCCACTACAATGG-3′ and 5′-GTGACTCCAGGCACCAGACT-3′), respectively.

Rapamycin, an inhibitor of mTORC1, was given to the *Tsc2*-KO mice from E18.5 to 3 months of age using a regimen that was effective in rescuing the lethality caused by neural *Tsc1* mutation without notable toxicity (36), i.e., intraperitoneal injection of rapamycin (1 mg/kg, LC Laboratories) every 4 days from E18.5 to P20, then 3 times per week (2 mg/kg) until the end of 3 months.

### Human BHD lung samples.

FFPE blocks from lung biopsies of 3 patients with genetically confirmed BHD were provided in-house after receiving approval by the University of Florida IRB. Normal human lung tissues were obtained from the local organ procurement office, LifeCenter, under the approval of the University of Cincinnati IRB.

### Histology and morphometric analysis.

Mice at different ages were weighed and euthanized. Following intratracheal cannulation, lung tissues were inflated under 25 cm H_2_O pressure, and different lobes were either fixed in 4% buffered paraformaldehyde or frozen and stored at –80°C. The fixed tissues were embedded in paraffin and sectioned with 5 μm thickness. These tissue sections were used for H&E staining or immunohistochemistry. For lung morphometric analysis, 5 sections of each tissue were randomly chosen at approximately 100 mm intervals. At least 5 lungs per genotype were analyzed at each time point. The MLI was used to measure average size of alveoli using Fiji software (37).

### Immunofluorescence staining and Western blot analysis.

The method for tissue immunofluorescence staining has been described previously (13). Briefly, lung tissue sections were boiled in Tris-EDTA buffer (pH 9.0) for antigen retrieval before incubating with the following primary antibodies: goat anti-PECAM1 (AF3628, R&D Systems), rabbit anti-SCGB1A1 and anti-SFTPC (WRAB-3950 and WRAB-9337, Seven Hill Bioreagents), mouse anti-TUBB4A (MU178-UC; BioGenex), hamster anti-PDPN (clone 8.1.1, DSHB at the University of Iowa), mouse anti-ACTA2 and mouse anti-cytokeratin (A2547 & C2562; Sigma), mouse anti-CDH1 (610182, BD Biosciences), and rabbit anti-phospho-S6 ribosome protein (2211, Cell Signaling Technology). Donkey secondary antibodies conjugated with Alexa Fluor 488, Alexa Fluor 594, and Alexa Fluor 647 were obtained from Thermo Fisher Scientific (A21202, A21207, and A21447). Fluorescence images were taken using the Leica Stellaris 8 at the Imaging Core Facility of the University of Cincinnati. More than 5 sections per sample were stained, and at least 5 randomly selected fields per section were imaged for evaluation.

Detection of proteins in tissue lysates by Western blot has been previously described (38). Proteins of interest were detected using the following specific antibodies: rabbit anti-GAPDH, pS6, S6, phospho-4E-BP1, 4E-BP1, RAPTOR, and mouse anti–β-actin (2118, 2211, 2217, 2855, 9644, 48648, 3700 from Cell Signaling Technology) and rabbit anti-FLCN antibody (a gift of Arnim Pause at McGill University, Montréal, Quebec, Canada) (39). Multiple independent mouse lung samples per genotype were analyzed.

### Total RNA isolation and RNA-sequencing analysis.

The middle lobe of the lung was harvested after exsanguination and flash-frozen in liquid nitrogen. mRNAs from WT and *Flcn* KO lungs were prepared by using the TRIzol RNA extraction reagent (Thermo Fisher Scientific, 15596026). The RNeasy Micro Kit (QIAGEN, 74004) was then used to clean up the extracted mRNA. Total RNA quality was checked with a Bioanalyzer (Agilent) with RNA integrity number greater than 7.0 being acceptable. RNA sequencing was performed at Novogene. Data processing was performed using the University of Southern California high-performance computing cluster (https://carc.usc.edu/). Roughly 50 million 100 bp, single-end sequences were aligned to the Ensembl 107 annotation based on the Genome Reference Consortium mouse genome (GRCm39) and using the STAR aligner (40, 41). Read counts per gene were calculated using the HTSeq-count software (42). Differential gene expression was determined using the R/Bioconductor software RUVSeq featuring edgeR (43). Genes were considered significantly different if their FDR-corrected *P* values were less than 0.05 and log_2_ fold-change was equal to or greater than 2.0. Venn diagrams and graphics were made using the software packages limma and ggplot2 (44, 45).

### Lung mesenchymal cell isolation and analysis for proliferation and apoptosis.

Lung mesenchymal cells were isolated and cultured using a method described in our previous publication (13). Briefly, after whole-lung tissue digestion, cells were cultured at low density (~10^4^/100 mm dish) for 2 weeks, and colonies of lung mesenchymal stem cells were isolated and replated as passage 1. The cell growth and apoptosis of the passage 2 culture were analyzed by EdU labeling (C10337, Thermo Fisher Scientific) and TUNEL assay (S7110, MilliporeSigma), respectively, following the methods described in our previous publication (46). The cells with additional treatment of DNase I served as the positive control for TUNEL assay.

### Statistics.

Multiple lung samples per genotype were collected at each time point for the experiments. The quantitative data are presented as mean ± SD. Statistical analyses were performed using 2-tailed independent-sample *t* test for 2-sample comparison and 1-way ANOVA for multiple-sample comparison (GraphPad Prism 10), with *P* < 0.05 considered significant.

### Study approval.

Animal studies followed the NIH Animal Research Advisory Committee Guidelines, and all procedures were approved by the Institutional Animal Care and Use Committees at Children’s Hospital Los Angeles and University of Cincinnati.

FFPE blocks from lung biopsies of 3 genetically confirmed BHD patients were provided in-house after receiving approval by the University of Florida IRB protocol (IRB202400482). Normal deidentified human lung tissues were obtained from the local organ procurement office, LifeCenter, under the University of Cincinnati IRB protocol number 2013-8157.

### Data availability.

The RNA-sequencing data were deposited to the NCBI GEO repository with accession number GSE266599. The graphical data are available in the Supporting Data Values file.

## Author contributions

Concept and design were developed by EPH and WS. KC, HC, LC, YL, HJW, JZ, JC, DK, NA, MET, and WS were responsible for conducting experiments and acquiring data. AA, NG, and KAWB were responsible for providing reagents and tissues. KC, DK, NA, MET, BHG, FXM, NG, KAWB, EPH, and WS were responsible for analysis and interpretation. EPH, NG, FXM, and WS were responsible for writing the manuscript. KC and HC are shared first authors based on their contributions to this work.

## Funding support

This work is the result of NIH funding, in whole or in part, and is subject to the NIH Public Access Policy. Through acceptance of this federal funding, the NIH has been given a right to make the work publicly available in PubMed Central.

- NIH/NHLBI grants R01 HL141352-01 (WS) and R01 HL146541 (WS & EPH).
- NIH/NCATS grant 2UL1TR001425 (University of Cincinnati, Center for Clinical and Translational Science and Training).

## Supplementary Material

Supplemental data

Unedited blot and gel images

Supporting data values

## Version 1 02/16/2026

Electronic publication
