# Supplementary material for: Hyperactivation of mTORC1 signaling mediates folliculin deficiency–induced pulmonary cyst formation in Birt-Hogg-Dubé syndrome
Source: J Clin Invest. 2026 Feb 16;136(4):e194300. doi: 10.1172/JCI194300 (PMC12904720; doi:10.1172/JCI194300)
Supplement: Supplemental data [file jci-136-194300-s009.pdf]

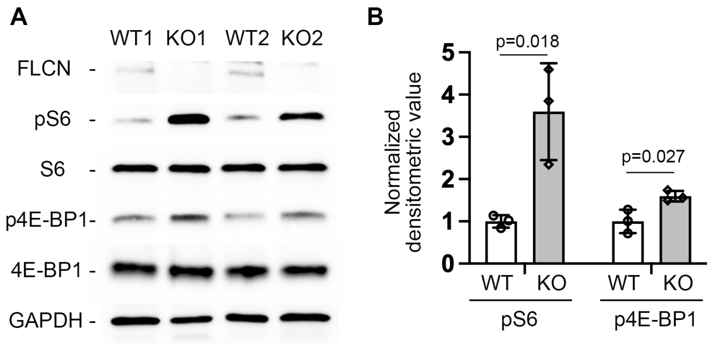

Supplemental Figure 1. Increased mTORC1 activity in Flcn-deleted lung mesenchymal cells. (A) Western blot detections of FLCN, phosphorylated and total S6 and 4E-BP1 in primary lung mesenchymal cells isolated from LM-*Flcn* knockout (KO) or wild type (WT) mice. GAPDH was used as a loading control. (B) Quantitative comparison of pS6 and p4E-BP1 levels from three biological replicates, normalized by total S6 or total 4E-BP1. p-value was calculated by unpaired T-test.

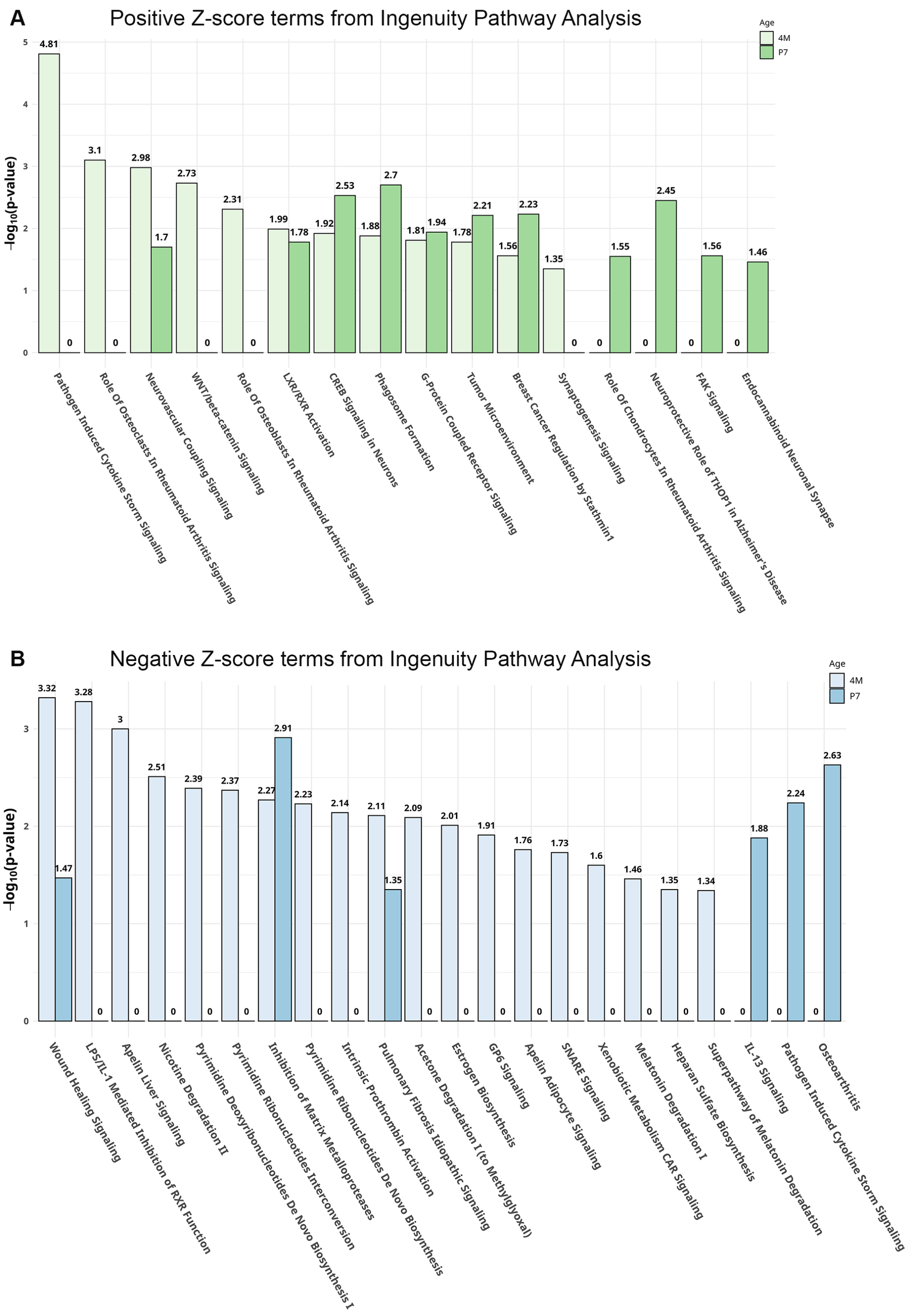

Supplemental Figure 2. Significantly activated or inhibited pathways in *Flcn* knockout lungs at P7 and 4 months of age, as predicted by Ingenuity Pathway Analysis ($-\log_{10}P>1.3$). (A) Terms with positive Z-scores are predicted to be activated. (B) Terms with negative Z-scores are predicted to be inhibited.
