# Supplementary figures and images for: Hyperactivation of mTORC1 signaling mediates folliculin deficiency–induced pulmonary cyst formation in Birt-Hogg-Dubé syndrome
Source: J Clin Invest. 2026 Feb 16;136(4):e194300. doi: 10.1172/JCI194300 (PMC12904720; doi:10.1172/JCI194300)

Figure 1A:

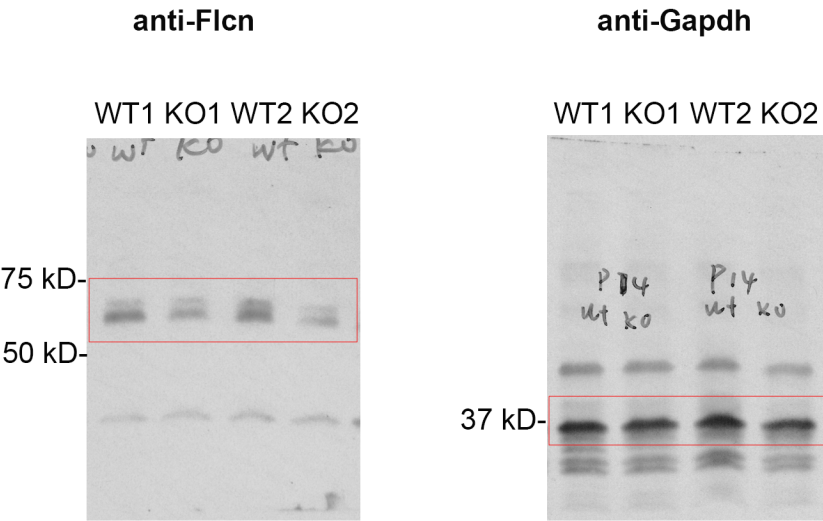

Figure 6A

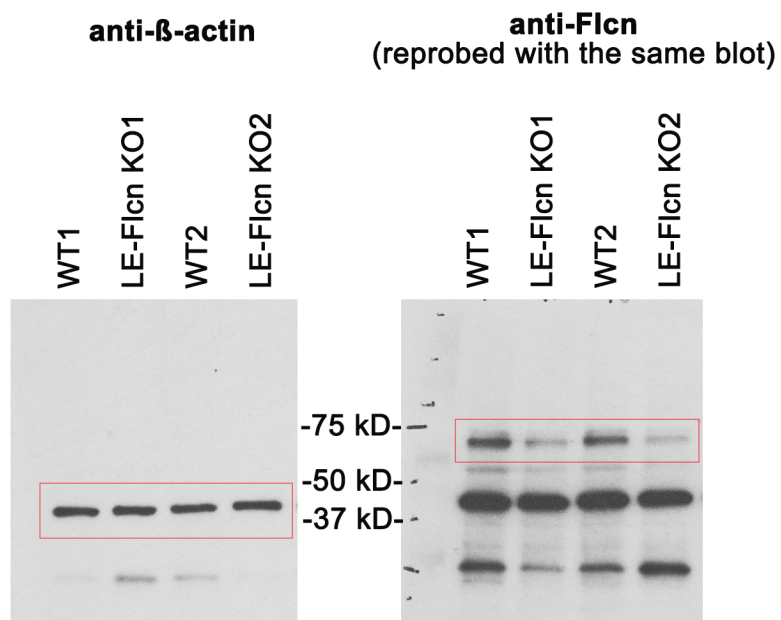

Figure 7B

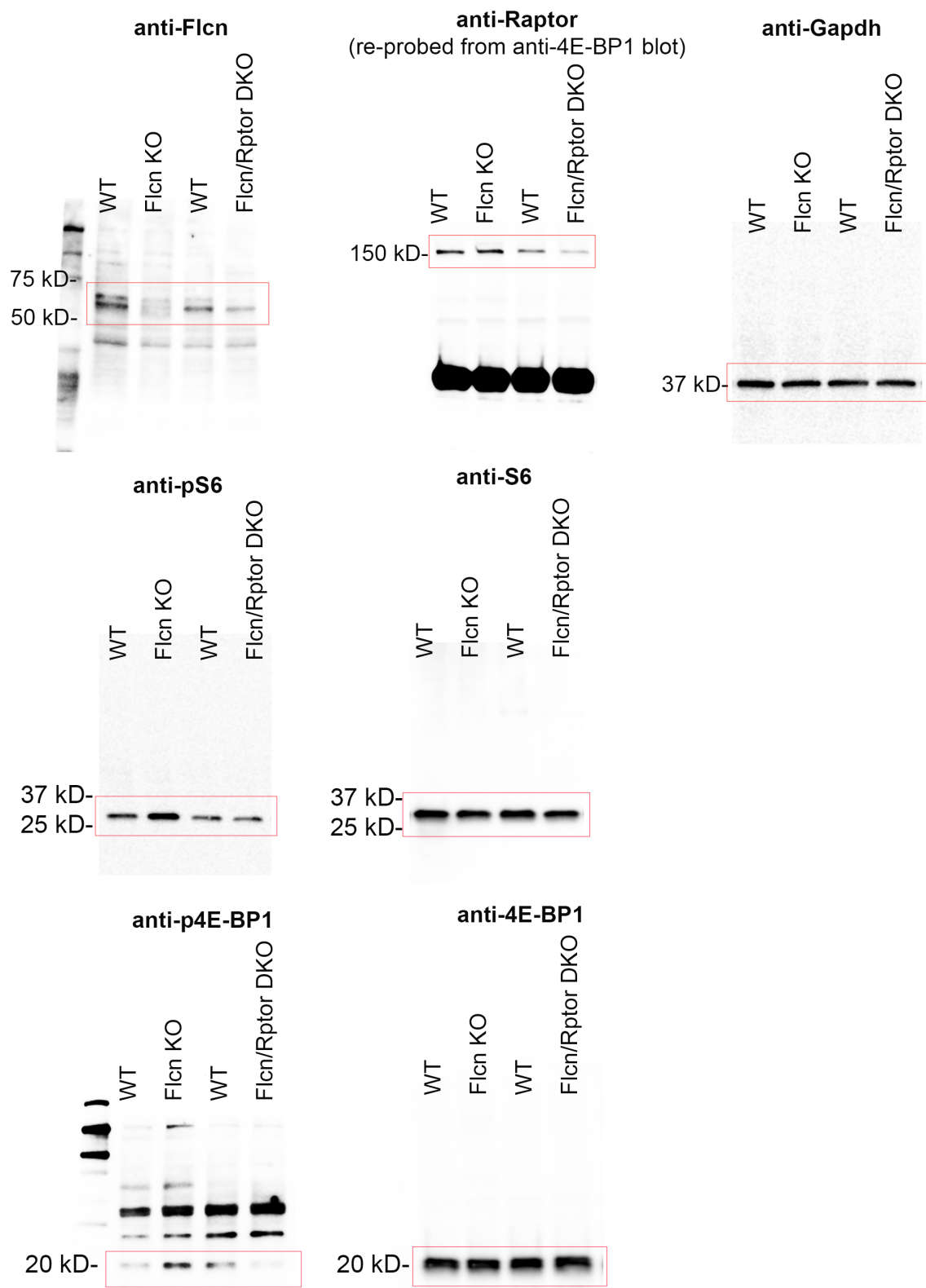

Supplemental Figure 1A.

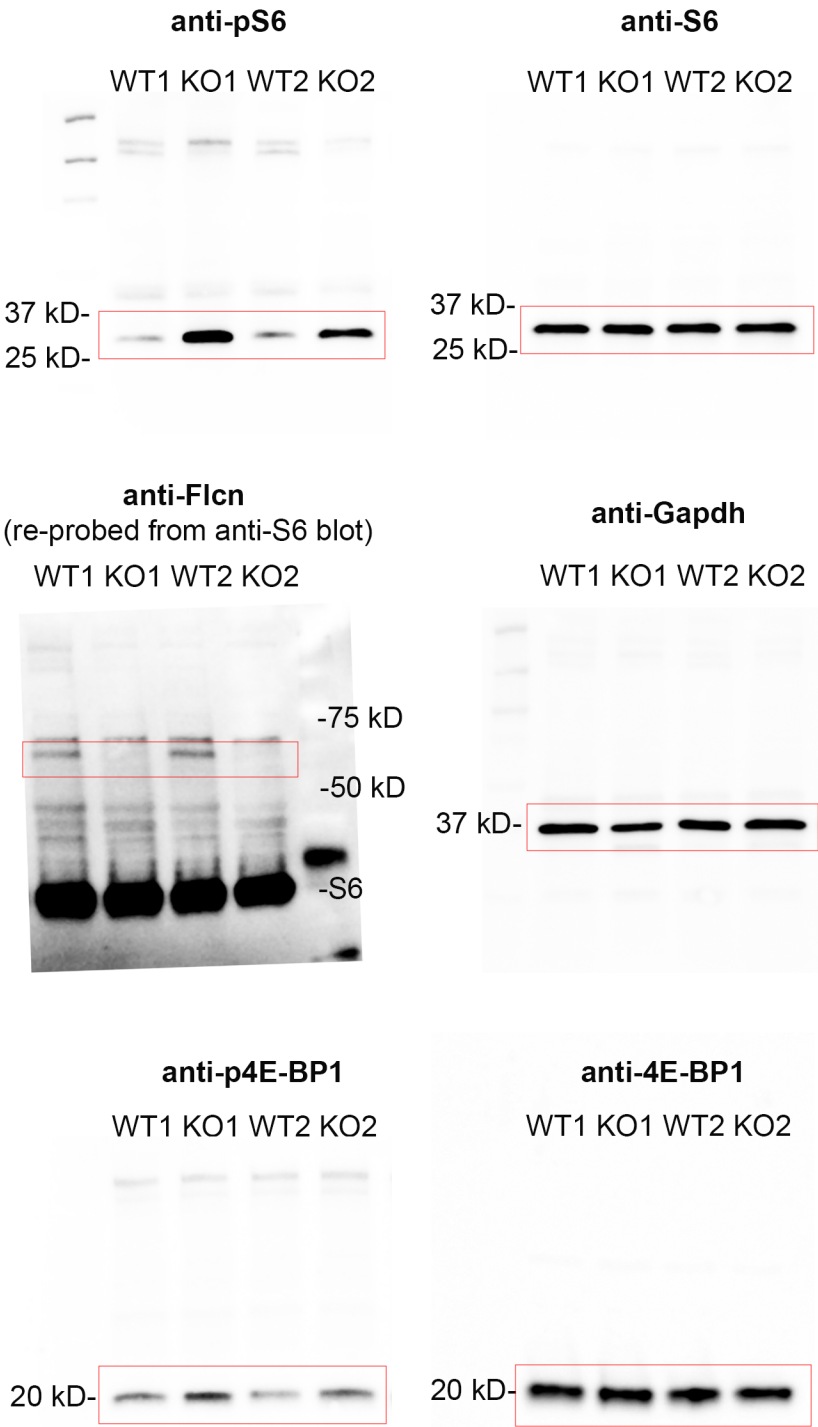

Supplement: Unedited blot and gel images [file jci-136-194300-s010.pdf]
